# Sudden Cardiac Arrest in Hemodialysis Patients with Wearable Cardioverter Defibrillator

**DOI:** 10.1111/anec.12119

**Published:** 2013-11-20

**Authors:** Chingping Wan, Charles A. Herzog, Wojciech Zareba, Steven J. Szymkiewicz

**Affiliations:** ^1^ Clinical Research ZOLL Pittsburgh PA; ^2^ Chronic Disease Research Group/Hennepin County Medical Center University of Minnesota Minneapolis MN; ^3^ Cardiology Clinical Research University of Rochester Medical Center Rochester NY

**Keywords:** hemodialysis hazards, sudden cardiac arrest, wearable cardioverter defibrillator, ESRD, mortality

## Abstract

**Background:**

The survival outcome following a sudden cardiac arrest (SCA) in hemodialysis (HD) patients is poor regardless of whether an event takes place in or out of a dialysis center. The characteristics of SCA and post‐SCA survival with HD patients using a wearable cardioverter defibrillator (WCD) are unknown.

**Methods:**

All HD patients who were prescribed a WCD between 2004 and 2011 and experienced at least one SCA event were included in this study. Demographics, clinical background, characteristics of SCA events were identified from the manufacturer's database. An SCA event was defined as all sustained ventricular tachycardia/fibrillation (VT/VF) or asystole occurring within 24 hours of the index arrhythmia episode. The social security death index was used to determine mortality after WCD use.

**Results:**

A total of 75 HD patients (mean age = 62.9 ± 11.7 years, female = 37.3%) experienced 84 SCA events (119 arrhythmia episodes) while wearing the WCD. Sixty six (78.6%) SCA events were due to VT/VF and 18 (21.4%) were due to asystole. Most SCA episodes occurred between 09:00 and 10:00 (RR = 2.82, 95% CI [1.05, 7.62], P < 0.0001), followed by the 13:00–14:00 time interval (RR = 2.22, 95% CI [0.79, 6.21], P = 0.006). Acute 24‐hour survival was 70.7% for all SCA events; 30‐day and 1‐year survival were 50.7% and 31.4%, respectively. Women had a better post‐SCA survival than men (HR = 2.41, 95% CI [1.09, 5.36], P = 0.03).

**Conclusions:**

The use of WCD in HD patients was associated with improved post‐SCA survival when compared to historical data.

## INTRODUCTION

The all‐cause mortality for U.S. end‐stage renal disease (ESRD) patients who are hemodialysis (HD) dependent is 196.6 per 1000 patient‐years from 2010 (prevalent patients). The single largest cause of death in HD patients is linked to arrhythmic mechanism. Sudden cardiac death (SCD) is responsible for approximately 26.5% of all‐cause mortality and is linked to 64% of all cardiac deaths.^1^

Several factors, such as age, diabetes, and coexisting heart disease, contribute to the vulnerability of dialysis patients to SCD.^2^, ^3^, ^4^ In addition, burdens from the HD treatment are also important in the genesis of cardiac arrhythmias.^5^, ^6^ The drastic fluid and electrolyte shifts, metabolic abnormalities and pH changes, which contrasts greatly to the smooth and continuous clearance provided by healthy kidneys, all predispose to arrhythmic events.^4^, ^6^, ^7^

At meantime, HD patients suffer abysmal outcomes after cardiac arrest.^3^, ^8^, ^9^, ^10^, ^11^, ^12^, ^13^ Even in the case of in‐center cardiac arrest, both immediate survival rate and survival to discharge among HD patients are very poor.^7^

The lifesaving effects of the wearable cardioverter defibrillator (WCD) were first documented in the WEARIT/BIROAD studies.^14^, ^15^ Chung et al. further showed effectiveness of antiarrhythmic treatment and survival benefits with the WCD.^16^ However, no similar study has been done in HD patients who are determined to be at high risk of life‐threatening tachyarrhythmia. Our objective was to (1) examine the characteristics of sudden cardiac arrest (SCA) experienced by HD patients during their WCD therapy; (2) assess the short‐, medium‐, and long‐term survival after SCA.

## METHODS

### Study Population and Data Sources

In this retrospective observational study, we included HD patients in the United States who wore a WCD from January 2004 to December 2011 and who experienced at least one SCA event while wearing the WCD. ESRD patients who had not initiated HD treatment at the time of WCD prescription were excluded from this study.

The WCD indication, demographics (age and sex), compliance, electrocardiogram (ECG), and events data were collected from a national postmarket database maintained by the manufacturer (ZOLL, Pittsburgh, PA, USA). A chart review was performed to identify the patients’ HD status, race, cardiac history, left ventricle ejection fraction (LVEF or EF), and comorbid conditions. Patients were followed from day 1 after SCA to either death or the date of analysis (February 9, 2012), whichever comes first.

All patients had signed consents to allow their data to be used for quality monitoring and research purposes. This study was performed in adherence with the guidelines of the Declaration of Helsinki.

### Compliance

The wearable cardioverter defibrillator (WCD) combines a long‐term arrhythmia monitoring system with an external automatic defibrillator. It is composed of a garment containing three self‐gelling defibrillation patch electrodes, two on the back and one in the front; four nonadhesive ECG electrodes connect to a monitoring unit that weighs about 0.77 kg. This device continuously monitors a patient's heart rhythm and can automatically deliver up to five posterior‐apex defibrillation shocks. The technical details of the WCD have been described in full elsewhere.^14^, ^16^ Compliance was measured as both the length of use in days and the average hours during a day that a patient's WCD was active and at least two electrodes were contacting the skin. End of use (EOU) reasons for discontinuing use of the WCD were obtained from either a physician's notes or a patient's call report.

### Statistical Analysis

Descriptive statistics were reported as means (±standard deviation), medians (25th, 75th percentile), or ranges for continuous variables and frequencies and proportions for categorical data. Wilcoxon rank sum test was used for continuous variables and chi‐square tests or Fisher's exact test if appropriate were used for categorical variables. Data was considered statistically significant if a 2‐sided P value <0.05. Analyses were performed using Microsoft Excel (v. 2010, Redmond, WA, USA) and SPSS statistical software (v.18, IBM, Inc. Armonk, NY, USA).

#### SCA Event and Post‐SCA Survival

For this study, an SCA event was defined as any sustained ventricular tachycardia (VT), ventricular fibrillation (VF) or asystole occurring within 24 hours of the index arrhythmic episode while a patient was wearing a WCD. ECG tracings were retrieved from the device recording. In addition, patient call reports were used to determine event locations, patient's neurological status and the immediate outcome post‐SCA. Medium (<2 months) and long‐term (1 year) mortality outcomes were determined from the social security death index (SSDI). Kaplan–Meier survival analysis and log rank test were performed to compare survival results between all patients and those who experienced VT/VF events only. Cox proportional hazards modeling was conducted for adjusted survival analyses among risk factors.

#### Temporal Analyses

The exact timing of each arrhythmia episode was determined from the device's internal clock. In this analysis, only a whole hour for each time stamp was recorded. For instance, if an episode occurred at 9:34 AM, it was coded as “9.” For multiepisode SCA events, a shock delivered longer than 5 minutes after the previous shock was considered as an independent episode and was included in the analysis.^17^, ^18^ Frequencies of such episodes were calculated for each 24‐hour interval as well as for one of the four temporal clusters: 9:00–14:59, 15:00–20:59, 21:00–02:59, and 03:00–08:59. The relative risk (RR) for each interval was calculated; chi‐square test was performed to determine significance.

## RESULTS

### Patient Population

A total of 75 HD patients experienced at least 1 SCA event while wearing the WCD. Nine ESRD patients were excluded due to no mention of HD treatment at the time of WCD prescription.

Table 1 shows the patients’ basic demographics and clinical characteristics. The majority of the patients had impaired LV function (81.3%) and cardiomyopathy (56.0%); 74.6% suffered from three or more comorbid conditions. Current infection was the primary reason (36.0%) that ICD was not recommended at the time of WCD prescription. Other reasons for ICD delay included: waiting period for primary prevention criteria (26.7%), high risk of infection or death (8.0%), waiting period for AV fistula maturity (4.0%), surgery contraindication (2.7%), previous ICD malfunction (2.7%), patient ICD refusal (1.3%), and unknown (18.7%). Mean days worn were 62.9 ± 73.1 days (range from 2 to 308 days) and average daily use was 18.9 ± 4.6 hours.

**Table 1:** Patient Characteristics

	Mean ± SD or n (%)			
	All patients	Female	Male	
Characteristics	(N = 75)	(N = 28)	(N = 47)	P value
Age (year)	62.9 ± 11.7	64.1 ± 12.8	62.2 ± 11.1	0.83
Race				0.77
African American	25 (33.3%)	10 (35.7%)	15 (31.9%)	
Caucasian	24 (32.0%)	9 (32.1%)	15 (31.9%)	
Other	1 (1.3%)	0 (0%)	1 (2.1%)	
Unknown	25 (33.3%)	9 (32.1%)	16 (34.0%)	
Indication for WCD				0.34
Primary prevention	21 (28.0%)	11 (39.3%)	10 (21.3%)	
Secondary prevention	26 (34.7%)	9 (32.1%)	17 (36.2%)	
ICD explant/malfunction	27 (36.0%)	8 (28.6%)	19 (40.4%)	
Others not specified	1 (1.3%)	0 (0%)	1 (2.1%)	
LVEF (%)	27.4 ± 12.5	24.6 ± 11.3	28.9 ± 13.0	0.33
≥35%	14 (18.7%)	4 (19.0%)	10 (25.0%)	0.41
Diabetes^a^	39 (54.9%)	16 (59.3%)	23 (52.3%)	0.56
Hypertension^a^	59 (83.1%)	22 (81.5%)	37 (84.1%)	0.78
Ischemic heart disease^a^	51 (71.8%)	15 (55.6%)	36 (81.8%)	0.02
Heart failure^a^	34 (47.9%)	10 (37.0%)	24 (54.5%)	0.14
Peripheral vascular disease^a^	22 (31.0%)	4 (18.5%)	17 (38.6%)	0.11
Arrhythmia^a^	37 (52.1%)	12 (44.4%)	25 (56.8%)	0.31

a Comorbidity information for four patients (three males and one female) was missing.

Abbreviations: SD = standard deviation; WCD = wearable cardioverter defibrillator; ICD = implantable cardioverter defibrillator; LVEF = left ventricle ejection fraction.

### SCA Events

**Figure 1:**
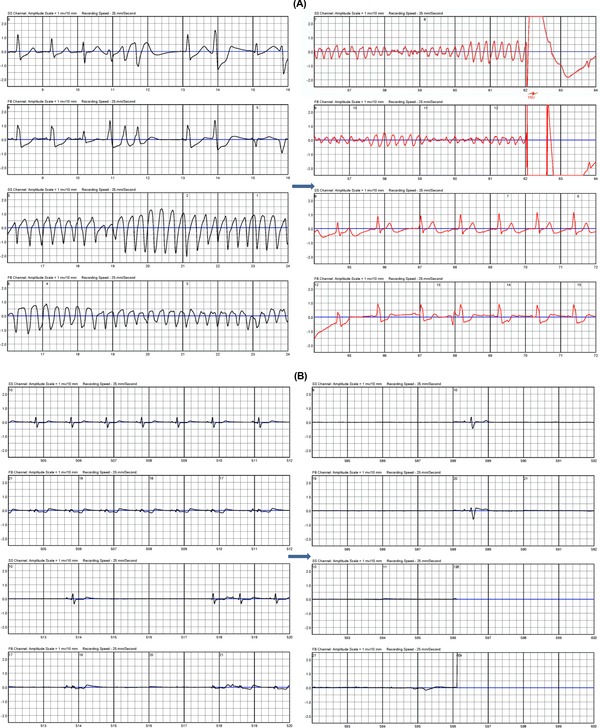
Example tracing for one VT/VF arrest (A) and one asystole event (B). VT = ventricular tachycardia; VF = ventricular fibrillation.

Seventy‐five patients experienced 84 SCA events (119 arrhythmia episodes); 66 events (or 101 arrhythmia episodes) by 57 patients were for VT/VF. No pulseless electrical activity was reported. WCD delivered a total of 136 shocks; the first shock conversion was 63 of 66 (95.5%). Eighteen patients experienced 18 asystole events; no shocks were delivered for asystole (Table 2). The WCD does not have pacing therapy to treat bradyarrhythmias; nonetheless, the WCD detects and broadcasts siren alerts and voice prompts for bystander assistance. Figure 1 shows two example tracings during a VT/VF arrest and an asystole event, respectively.

**Table 2:** Characteristics of the SCA Events (N = 84)

Characteristics	N (%)
Initial rhythm	
VT	54 (64.3%)
VF	12 (14.3%)
Asystole	18 (21.4%)
Location of event occurrence	
Home	30 (35.7%)
Hospital	13 (15.5%)
Dialysis unit	23 (27.4%)
Rehab/Nursing home	1 (1.2%)
Others	1 (1.2%)
Unknown	16 (19.0%)

VT = ventricular tachycardia; VF = ventricular fibrillation.

### Circadian Variation

**Figure 2:**
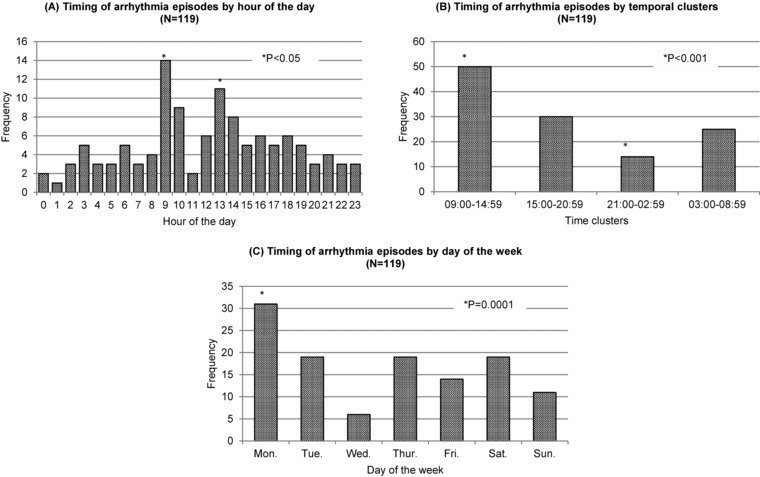
Timing of arrhythmia episodes by hour of the day (N = 119) (A). Timing of arrhythmia episodes by temporal clustering (N = 119) (B). Timing of arrhythmia episodes by day of the week (N = 119) (C).

Figure 2A shows the hourly frequency of observed independent arrhythmia episodes (n = 119). Fourteen episodes were excluded from the analysis due to that they occurred within 5 minutes from the previous one. The majority (72/119) of the episodes happened during the daytime between 09:00 and 18:00; of these, 35 (70.0%) occurred during dialysis sessions and 2 (2.8%) immediately after. The distribution was slightly skewed. It peaked at the interval of 09:00–10:00 (RR = 2.82, 95% CI [1.05, 7.62], P < 0.0001), followed by a slightly lower peak at the interval of 13:00–14:00 (RR = 2.22, 95% CI [0.79, 6.21], P = 0.006). The event episodes were more evenly distributed during the night time and early morning hours.

Results from clustering analysis are shown in Figure 2B. Similar to the hourly distribution, arrhythmia episodes tended to occur in temporal clusters (P < 0.0001). Most episodes occurred between 09:00 and 14:59 (42.1%, RR = 1.68, 95% CI [1.15, 2.45], P < 0.0001), followed by 15:00–20:59 (25.2%, P = 0.96) and 03:00–08:59 (21.0%, P = 0.31) intervals. There was least number of episodes occurring between 21:00–02:59 (11.8%, RR = 0.47, 95% CI [0.52, 1.34], P = 0.0009).

Figure 2C illustrates the timing of arrhythmia episodes by days of the week. It did not distribute evenly during the week. Monday was the day where most arrhythmias occurred (n = 31), follo‐wed by Tuesday, Thursday, and Saturday with 19 each; Wednesday had the least number of SCA arrhythmias (n = 6). The distribution was statistically significant (P = 0.001).

### SCA Survival Outcome

Acute 24‐hour survival was 70.7% (53 of 75 patients) for all SCA events. Among the survivors, 46 (86.8%) continued wearing the device for extended time period (mean = 31.0 ± 38.8 days, range from 1 to 145 days); 9 had a second event and 19 (41.3%) eventually received an ICD with an average 20.9 ± 36.7 days of extended wear. Other EOU reasons included: 11 deaths (23.9%), 2 (4.3%) with EF improved, 5 (10.9%) with condition deteriorated, and 7 (15.2%) with unknown reasons. Two (4.3%) were still using the device at the time of analysis.

**Figure 3:**
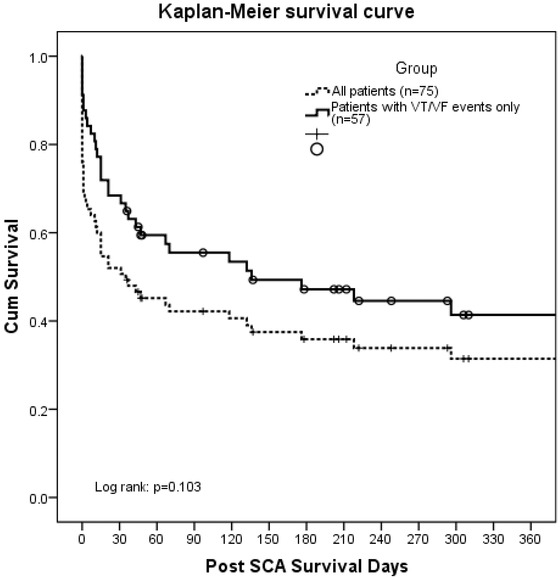
Kaplan–Meier survival curve.

Medium and long‐term Kaplan–Meier survival curves for VT/VF events compared to that for all events are shown in Figure 3. Among the 75 patients, 15 patients were censored due to not reaching the mortality endpoint. Median follow‐up was 35 (626.0, 1.0) days. Overall, the 30‐day survival for was 50.7% (38 of 75 patients); five additional deaths occurred between day 30 and day 60, making a 45.2% 2‐month survival. Post‐SCA mortality was the highest within 60 days. The 1‐year survival rate was 31.4%. The patients with VT/VF alone had better survival (91.2% within 24 hours) than those with asystole events in which only 1 patient survived more than 3 days post‐SCA.

**Figure 4:**
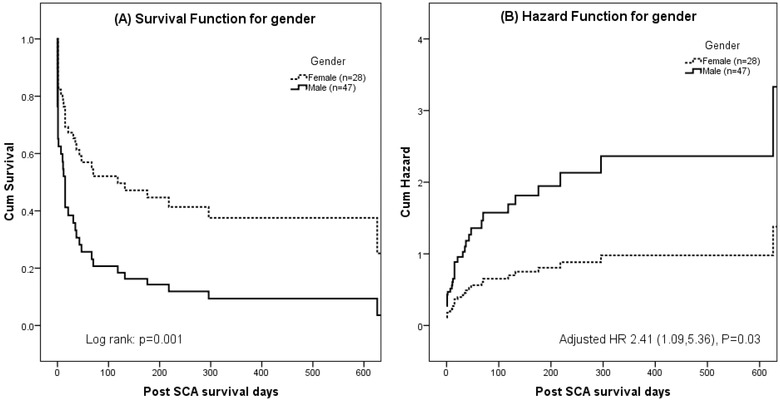
Survival curve (A) and hazard function (B) by gender.

Results from Cox proportional hazards analyses were shown in Table 3. Hazard ratio (HR) was adjusted for the type of arrhythmias experienced. Male gender was associated with increased risk of mortality (HR = 2.41, 95% CI [1.09, 5.36], P = 0.03). Median survival for male patients was 11.0 (132.0, 0) days, compared to 296.0 (725.0, 15.0) days for female group. The two were largely comparable, except that more men suffered from ischemic heart disease than women (81.8% vs. 55.6%, P = 0.02). Male group appeared to experience more asystolic events (34.0% vs. 10.7%, P = 0.04). Figure 4 shows the survival curves and hazard function between male and female groups. All other risk factors were not statistically significant.

**Table 3:** Cox Proportional Hazards Analyses in all Patients

	Hazard	95%	
	Ratio	Confidence	
Risk Factors	(adjusted)	Interval	P Value
Male gender	2.41	1.09–5.36	0.03
Age (year)	0.99	0.96–1.01	0.31
Previous ICD	0.71	0.36–1.42	0.34
Diabetes mellitus	1.78	0.90–3.53	0.10
Hypertension	0.43	0.17–1.08	0.07
Heart failure	1.64	0.78–3.44	0.19
Ischemic heart disease	1.07	0.48–2.41	0.86
Peripheral vascular disease	0.86	0.42–1.76	0.68

ICD = implantable cardioverter defibrillator.

## DISCUSSION

The risk of SCD among HD patients attributes to about a quarter of the total mortality.^1^ Compared to the general population, the incidence of SCD in HD patients is more than 50 times greater at approximately 53 deaths per 1000 patient‐years^1^ compared to the 1 death per 1000 patient‐years in the general population.^19^, ^20^ Although the survival rate following an SCA in the general population is low,^18^ HD patients face even worse outcomes. Moss et al. observed 6 of 74 (8%) dialysis patients survived to hospital discharge after cardiopulmonary resuscitation.^8^ Lai et al. reported a 0% post‐SCA survival until discharge, even with a 75% successful resuscitation rate and 45.8% survival rate at 24 hours.^10^ A retrospective study by Herzog et al. analyzed a sample of Medicare database and found that only 7853 dialysis patients survived at least 30 days from the index admission out of a total 30,230 who experienced cardiac arrest, making a 26.0% 30‐day survival; and among the same study cohort, 23.6% (7139 of 30230) survived until discharge.^3^ Three other studies reported a 30‐day survival as 19%^11^, ^12^, ^13^ and 1‐year survival as 9–15%.^3^, ^11^, ^12^

Previous studies show that defibrillators are often underutilized in the dialysis population. AEDs in dialysis units did not improve survival, since in only half of the cases was the AED attached prior to EMS arrival.^9^, ^21^ ICDs are not routinely prescribed for HD patients, possibly due to the potential complications or the lack of randomized clinical trial data supporting these considerations.^22^ The same retrospective study by Herzog et al. reported that of the 6042 dialysis patients eligible for ICDs, only 7.6% received one.^3^ In our study, active infection was listed as the top reason that an ICD was not recommended, followed by high risk of infection, poor prognosis, and surgery contraindication associated with vascular access.

Compared to the existing literature, we observed improved medium and long‐term survival post‐SCA (50.7% for 30 days and 31.4% for 1 year) among the study population. The WCD demonstrated promising therapeutic outcomes, especially for VT/VF arrest with a 91.2% acute survival rate. In addition, the majority of the event survivors continued wearing the device for extended period until EF improvement or a permanent solution (e.g., ICD) was available. The survival rate for asystolic arrest was poor but expected, given the reliance on bystander response and intervention. Compared to the historical data listed above, the utilization of WCD resulted in an absolute reduction of 30‐day mortality rate by approximately 30% and 1‐year mortality rate by 19%.

Male gender had a negative influence on survival outcomes. This was possibly due to that more men suffered from ischemic heart disease than women in the study group. This concurs with the findings by Chugh et al. in a prospective investigation regarding sex‐based differences in prevalence and manifestation of SCA, which concluded that women were significant less likely to have LV dysfunction and recognized coronary artery disease before SCA.^23^ Explanations to such difference in the prevalence of structural heart disease are multifold. For instances, level of estrogen, life‐style (i.e., smoking and drinking), disease management, prevalence of diabetes, and hypertension can all contribute to the vascular lesions. However, Chugh et al. did not observe a difference in rates of SCA survival to hospital discharge. It should also be noted that when adjusting the hazards model with types of arrhythmias, in particular asystole, the P value of gender risk factor increased from 0.002 to 0.03; while other covariates such as history of heart failure and arrhythmia were no longer statistically significant. As expected, asystolic events were associated with worse survival outcome; and in our group, men did experience slightly more asystole than women, although bias from small sample analysis cannot be ruled out.

Our study also observed a temporal pattern of arrhythmia episodes. Dialysis sessions are typically scheduled either on Monday, Wednesday, and Friday (MWF), or on Tuesday, Thursday, and Saturday (TTS) for the vast majority of U.S. HD patients. We observed that arrhythmias peaked on Monday which reflected the 12‐hour period starting dialysis session for a MWF schedule or the 12‐hour period before dialysis session following the weekend gap for a TTS schedule. The same trend was observed in other studies which reported that Mondays and Tuesdays were the most common days of SCD.^5^, ^6^, ^7^ In our results, Tuesday did have a cluster of episodes but did not outnumber those occurring on Thursdays and Saturdays. This might be due to a disproportionate number of patients having a MWF dialysis schedule versus a TTS schedule in our study cohort. Such disproportion was found by one earlier study which reported that 59.2% of patients dialyzed on MWF schedule versus 40.8% on TTS schedule.^24^ However, the exact distribution could not be determined as the information was not always available on the patient's cardiology progress notes.

The WCD's internal clock allowed us to record the exact time stamps of each independent arrhythmia episode. We observed that the event rate peaked between 09:00 and 14:59 with more than 50% of increased risk. The risk went down as time proceeding to the evening. The event rate reached the lowest point during the 21:00–02:59 interval. Such circadian variation was in accordance with conventional daytime HD sessions; and this is very different from the early‐morning peak of SCD in patients with ischemic heart disease. In addition, a bimodal distribution of hourly event rate was observed, with a 2.82 increased risk of arrhythmia during the 9:00–10:00 interval and a 2.22 increased risk during 13:00–14:00 interval. The 1‐hour block next to each of the above‐mentioned intervals had high incidence of SCA as well. It was not surprising that the occurrences of arrhythmia flattened out during the late night and gradually picked up during the early morning hours, as fewer nocturnal dialysis sessions are offered in most dialysis units. Both findings confirmed that SCA occurred more often around the dialysis sessions. This aligns with what Bleyer et al. found in a retrospective study involving 80 SCD in HD patients.^5^ The contributing factors are multifold. The high prevalence of LV dysfunction, ischemic heart disease, and heart failure in our study population provided a substrate for the arrhythmias to occur. In addition, during the typical 4‐hour dialysis session, fluid shifts and electrolyte imbalance, such as potassium fluxes, could lead to arrhythmic events. Predialysis volume overload often results in hyperkalemia and hypertension; rapid removal of fluid during the dialysis leads to postdialysis hypotension and hypokalemia. These situations are commonly seen and having disastrous consequences, as both hyper and hypokalemia are well‐known risk factors for SCD.^7^, ^25^, ^26^, ^27^, ^28^ Different dialysate may have an impact on SCA risk as well. Karnik et al. reported that patients with a 0 or 1.0 mEq/L potassium dialysate arrested later in the course of dialysis than patients on a 2.0 or 3.0 mEq/L potassium dialysate.^7^ Avoidance of low‐potassium dialysate (<2.0 mEq/L) may be a modifiable risk factor for the prevention of SCD in dialysis patients.^11^ Morrison et al. demonstrated decreased ventricular ectopic activity in 4 of 6 patients whose dialysate potassium level changed from 2.0 to 3.5 mEq/L.^25^

Our study provides compelling results that the WCD may reduce SCD among the HD patients who are complicated with various cardiac diseases. However, it has two limitations. First, clinical information such as serum potassium levels, dialysate prescription as well as the dialysis session schedule were not always reported, because the medical charts faxed to the manufacturer were for insurance purposes and were sometimes limited to the cardiology progress notes. Second, our study cohort did not have a control group: who were using the WCD but did not have an event or who had an event but not a WCD patient. Although these limitations should not change our conclusion, such information may strengthen our findings regarding the survival benefits rendered by the WCD as well as the impact of different variables on the SCA risk.
